# Applications of molecular networks in biomedicine

**DOI:** 10.1093/biomethods/bpz012

**Published:** 2019-09-23

**Authors:** Monica Chagoyen, Juan A G Ranea, Florencio Pazos

**Affiliations:** 1 Computational Systems Biology Group, Systems Biology Program, National Centre for Biotechnology (CNB-CSIC), Madrid, Spain; 2 Department of Molecular Biology and Biochemistry, University of Malaga, Malaga, Spain; 3 CIBER de Enfermedades Raras, Instituto de Salud Carlos III, Madrid, Spain

**Keywords:** biological networks, systems medicine, human pathologies

## Abstract

Due to the large interdependence between the molecular components of living systems, many phenomena, including those related to pathologies, cannot be explained in terms of a single gene or a small number of genes. Molecular networks, representing different types of relationships between molecular entities, embody these large sets of interdependences in a framework that allow their mining from a systemic point of view to obtain information. These networks, often generated from high-throughput omics datasets, are used to study the complex phenomena of human pathologies from a systemic point of view. Complementing the reductionist approach of molecular biology, based on the detailed study of a small number of genes, systemic approaches to human diseases consider that these are better reflected in large and intricate networks of relationships between genes. These networks, and not the single genes, provide both better markers for diagnosing diseases and targets for treating them. Network approaches are being used to gain insight into the molecular basis of complex diseases and interpret the large datasets associated with them, such as genomic variants. Network formalism is also suitable for integrating large, heterogeneous and multilevel datasets associated with diseases from the molecular level to organismal and epidemiological scales. Many of these approaches are available to nonexpert users through standard software packages.

## Introduction

The reductionist approach of molecular biology was very successful in biomedicine. Getting insight into the molecular mechanisms underlying pathological processes allowed to use molecular entities (e.g. genes, proteins and metabolites) as markers for diagnosing diseases or targets for treating them. Nevertheless, biological systems are the prototype of “complex systems,” as they are characterized by a large number of molecular components immersed in intricate networks of interactions. As such, many of their properties resist a reductionist approach and can only be tackled from a systemic point of view [1–5].

Disease-related phenomena are not an exception, and the reductionist approach has clear limitations in the case of complex diseases involving a large number of “causative” or associated genes, such as cancer or Alzheimer’s disease [6]. The reductionist approach to diseases assume that they can be tracked back to a reduced number of molecular entities, whose effects on the pathology are fundamentally additive, so that studying them in isolation and later combining the results in a simple way would allow for understanding the disease at molecular level. Even in prototypical monogenic diseases, for which the reductionist approach is expected to work well, the causative gene(s) do not work in isolation but are immersed in large molecular networks. Consequently, even if the onset of a disease depends on a single gene, other important factors, such as its severity or patient-specific manifestation, depend on many other genes/mutations, requiring a more systemic approach for understanding them (e.g. cystic fibrosis [7]). The partial failure of promising therapeutic approaches such as peptide vaccines, genetic therapy, antisense RNA or rational design of vaccines is partially attributed by some authors to their extreme reductionist basis [4, 8–10]. The limitations of reductionist approaches to diseases could also partially explain the continuous reduction of new drugs brought to the marked in spite of increased inversion [11], as the current model of drug-development has a strong reductionist basis.

Systemic approaches to biological phenomena focus on the complex networks of interactions between molecular components instead of the detailed properties of components themselves [1–5]. These approaches were delayed in part by the lack of large data required to build these networks. These datasets are now generated by omics approach, and the networks assembled from them are the prototypical subject of study of molecular systems biology [12–16]. These systemic approaches are applied to the study of human pathologies, what is sometimes called Network or Systems Medicine. The new evidence and results derived from the application of systems medicine are driving a paradigm shift in both the identification of new therapeutic targets and the development of drugs, leading to the emergence of new disciplines such as network pharmacology [17] and polypharmacology [18, 19].

In this review, we try to provide an introductory overview of this emerging field. Since this is a hot topic and the field is fast-moving, it is difficult to assemble a comprehensive summary that fully covers the subject. Consequently, we focus on the concepts that are well-established and form the basis of these methodologies. We start by providing an overview on the main molecular networks, focusing on their main organizing principles and their relationship with pathologies. Then we summarize the main approaches for extracting disease-related information from these networks. We pay special attention to the practical application of these approaches, including information for interested users on how to use these approaches through standard software packages.

## Molecular networks

**Figure 1:**
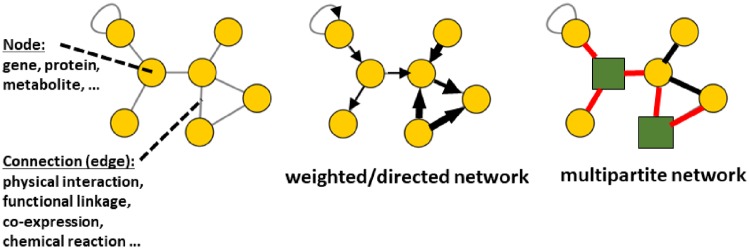
Left: Schematic representation of a small network with seven nodes and eight edges. Middle, a similar network with edge weights and directionality (directed/weighted network). Right: A multipartite network with two different kinds of nodes (yellow circles and green boxes) and two types of edges (black and red).

A network (or graph in mathematical terms) is a representation of a set of relationships between entities. Any phenomenon that can be described in terms of entities linked by relationships can be modeled as a graph. The entities are usually called nodes or vertices, and the relationships are edges, links or connections (Fig. 1). A node can represent a definite physical object (e.g. protein, metabolite, person, computer, etc.) or concepts that are more complex (e.g. cell type, developmental stage, disease, software subroutine, etc.). Likewise, edges can represent any type of linkage between nodes (physical interaction between proteins, chemical transformation between metabolites, hypertext link between two web pages, subroutine call in a computer program, etc.). Consequently, nodes and edges represent entities and relationships understood in the broadest sense. The relationship represented by the edges can be either directed, e.g. a chemical transformation of a compound into another, or undirected, e.g. a physical interaction between two proteins. Similarly, the edges can have different associated values (weights), representing a quantitative property, or all edges can have the same value. Consequently, according to the nature of their edges, networks can be classified as directed/undirected and weighted/unweighted (Fig. 1). Some networks are not homogeneous in terms of the entities represented by their nodes and edges. If the nodes (or edges) represent different types of entities, the network is multipartite (Fig. 1). All these characteristics (weights, direction, different types of nodes, etc.) depend on the nature of the phenomenon we are modeling as a network.

Once we have a given phenomenon/dataset represented as a network, we can mine it using the tools of Graph Theory. These approaches quantify topological network parameters that can have a translation into properties of the phenomenon modeled in that network, hence providing information on it.

Phenomena involving large datasets with intricate relationship patterns are particularly suitable for network representation. Consequently, different datasets across diverse disciplines have been modeled as networks. Network approaches are very popular for analyzing “social networks,” where the nodes represent persons and the edges represent some type of social linkage, such as friendship. Technological networks such as the World Wide Web are also frequently subjected to this kind of representation.

In Biology, this approach has been commonly used for representing ecological relationships, such as predator–prey linkages (“food webs”), as there are enough data associated with them. Molecular networks (i.e. those representing relationships between molecular entities or any other phenomena at molecular level) are more recent, as the large datasets required to assemble them are recent too. These datasets usually come from “omics” techniques that allow retrieving large amounts of molecular information in a massive way, part of which can be represented as networks [13, 20].

The main molecular network is the “interactome,” in which the nodes represent proteins and the (undirected) edges represent interactions between them [21]. The advent of high-throughput experimental methods for detecting protein interactions, in combination with methods for predicting interactions and relationships from genomic information or for mining the literature in search of described relationships, has made it possible to assemble large interactomes for most model organisms. In many cases, these diverse “evidences” of interaction are combined in different ways to obtain an interactome with high reliability and coverage [22]). Especially for human protein interactions, it is a common practice to use gene expression data to instantiate a generic interactome obtained by cell-free, high-throughput methods into that taking place at a particular tissue or cell type [23].

**Figure 2:**
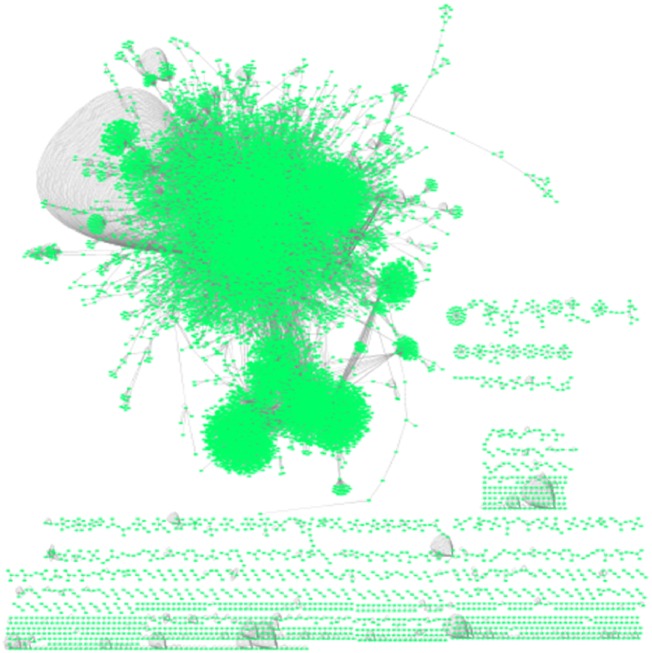
Undirected and unweighted network representing the human protein–protein interactions stored in the BIND database [24]. Representation is generated with Cytoscape [25]. The network comprises 19 905 nodes (proteins, green) and 38 706 edges (interactions, gray).

Figure 2 shows the human interactome stored in the BIND database [24].

Another molecular phenomenon usually represented as a network is cellular metabolism. In a typical representation of a metabolic network, the nodes are chemical compounds (metabolites) and the (directed) edges represent chemical transformations from one metabolite to another [26]. These metabolic networks have been assembled mainly with the data accumulated throughout decades of detailed experimental characterization of biochemical reactions as well as by homology-based metabolic reconstruction. Sometimes, the metabolism is also represented as a bipartite network with two types of nodes, metabolites and enzymes. In this representation, the directed edges represent enzyme-product (enzyme → metabolite) and enzyme-substrate (metabolite → enzyme) relationships. An enzyme-centric representation in which nodes are enzymes and edges represent “consecutive” enzymes (i.e. the product of one enzyme is substrate for the other) is also used widely.

The “Regulome” is a network representation of the gene regulatory relationships taking place in a given organism. In this network, nodes represent genes and the directed edges represent relationships between transcription factors and their regulated genes [27]. Besides direction, edges in a regulome should also contain information on whether the transcriptional control is for activation or repression. This can be done either using weighted edges (e.g. 1 for activation and –1 for repression) or through a bipartite network with two types of edges, representing activation and repression relationships.

While these are the three major molecular networks, many others have been assembled and studied. For example, “genetic networks” are undirected networks representing genetic associations between genes: genes for which the phenotypic effects of their mutation are not independent [28, 29]. The “phosphorylome” is the (directed) network of associations between kinases and their protein substrates [30, 31]. Co-expression networks are undirected networks where nodes are proteins and edges link pairs of proteins with similar expression patterns across a set of experiments [32].

The tendency is to combine many of these networks in multipartite networks [33]. For example, metabolic, gene regulation and protein interaction data can be combined and represented as a multipartite network with two types of nodes (metabolites and proteins) and three types of linkages: directed reactions between metabolites, undirected interactions between proteins, and directed regulatory relationships between proteins.

## Network approaches to diseases

Network medicine can be defined as systemic approaches to study and treat human diseases. These approaches complement the classic reductionist approach of molecular biology based on trying to understand a disease as caused by a problem in one or a very small number of genes that can therefore be treated by a single drug (“1 disease–1 gene–1 drug” paradigm). Systemic approaches use a more holistic approach based on the idea that diseases are better reflected at higher levels of complexity involving many molecular entities as well as environmental factors entangled in complex relationships. Network approaches to diseases use networks to represent such complex and large sets of molecular entities and their diverse relationships, and graph theoretical and related methods to extract disease-related information from these [16, 34–38]. While in the reductionist approach individual genes and/or proteins are markers and eventual therapeutic targets for diseases, in the systemic/network approach, markers and targets are large (sub)networks. The idea is that most diseases are better reflected at the level of complex networks instead of single genes. Accordingly, diseases arise as emergent properties of complex networks, which are affected by both genetic and environmental factors [39]. Diseases are seen as perturbations in the network structure (e.g. rewiring) more than in the nodes (genes) themselves [16].

In principle, all the molecular networks described in the previous section, representing different types of linkages between diverse molecular entities, as well as multipartite networks that combine them, can be used in these systemic approaches to study diseases. The different capacity of these diverse molecular networks for assisting in the discovery of disease genes has been benchmarked in some cases [40].

## Disease-related genes and network modules

The most obvious way in which network information can be used to obtain disease-related information is to look into protein networks for topological features of the nodes associated with disease. The degree of a node (number of connections) is a very simple topological parameter, and in protein networks it has been shown to be related to the “importance” of the corresponding gene/protein for the organism. For example, highly connected nodes (sometimes called “hubs”) tend to correspond to essential genes [41] and to be conserved evolutionarily [42]. Another topological parameter related to node importance is “betweenness.” The betweenness of a node is the number of internode shortest pathways that requires that particular node. Nodes with high betweenness (sometimes called “bottlenecks”) also tend to be essential genes, especially in directed networks such as the regulome [43]. So, both hubs and bottlenecks would be good candidates for disease-related proteins due to their importance for the functioning of an organism. Indeed, it has been shown that proteins involved in diseases tend to have more interactors than average [34, 44–46] and higher betweenness [45]. Nevertheless, even if more connected than the average, disease-related proteins are not the hubs of interactomes [47]. The reason is that hubs are essential and hence their failure would not lead to a viable organism that can manifest disease. Moreover, these topological properties of disease-associated genes are dependent on the characteristics of the disease (Mendelian, somatic, monogenic, dominant/recessive, etc.) [48].

**Figure 3:**
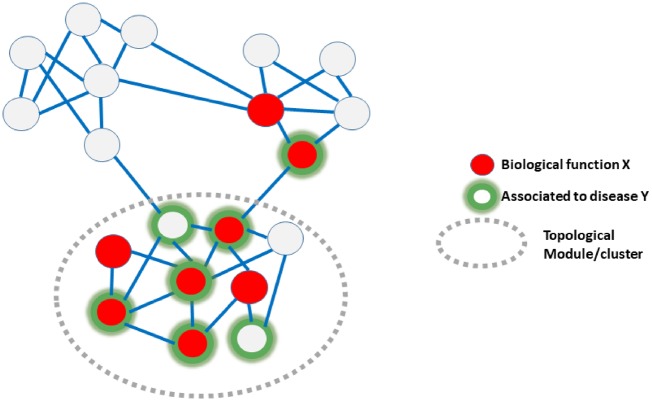
Relationships between topological, functional and disease-related modules. The schematic network has three topological modules. The proteins involved in a specific function (“X”) are colored red. Those associated with a given disease (e.g. those whose mutation is known to cause “disease Y”) are highlighted with green halos. Proteins known to be involved in “function X” tend to cluster in the network. Those associated with disease Y also tend to cluster in the same topological module, indicating that disease Y may be related to a dysfunction of the biological process X. Mutations of other proteins in the same topological/functional module would probably cause the disease as they disrupt the same process, but they have not yet been detected.

A key concept in network medicine is that of “disease-related module.” A module (also known as “cluster” or “community”) is a group of nodes highly connected among themselves but sparsely connected with the rest of the network (Fig. 3). As such, “module” is a purely topological concept. In molecular networks, these modules have been shown to comprise functionally related molecules [49]. For example, a set of interacting proteins involved in the same biological process and/or forming a macromolecular complex such as the ribosome or the proteasome forms a topological cluster in the interactome. Similarly, topological clusters detected in metabolic networks are in good agreement with the traditional metabolic pathways defined by expert knowledge and grouping-related metabolites [50]. Hence, topological modules within molecular networks are also functional modules (Fig. 3) in the sense that a given topological module can be associated with a given biological function. This allows, e.g. to infer the function of nodes using the principle of “guilt by association” [51, 52], i.e. a node of unknown function immersed in a module/cluster would probably share a role with the rest of the module. As genes with similar functions cluster in molecular networks, so do genes associated to the same disease (Fig. 3). This has been shown in multiple global studies as well as in those focused in particular diseases [53–56]. In a disease such as cancer, characterized by the progressive accumulation of mutations that at some point triggers the manifestation of pathology and other phenotypic transitions, it has been shown that it is the clustering of these mutations in defined network modules which is associated with these transitions, and not the mere accumulation of mutations all through the network [54]. Even in very complex diseases involving hundreds/thousands of genes, these are not spread through the network but tend to concentrate in a reduced number of modules/pathways (e.g. autism [57]). Not only the genes associated with a given disease cluster in molecular networks but those associated to a given symptom or patho-phenotype (e.g. fever, hemorrhage, inflammation, seizures, etc.) concentrate in networks as well [58]. Consequently, we can define a “disease module” as a topological network module associated with a disease, i.e. a network module that contributes to an abnormal phenotype associated with that disease when its components (nodes) are dysfunctional [35].

## Locating disease-related modules

Disease-related modules are usually identified from an initial set of “seed” genes associated with a given disease. These can be genes whose mutation is known to cause the disease, genes accumulating variants in observational studies such as “genome-wide association studies” (GWAS), or genes/proteins “altered” in some way during the manifestation of the disease (e.g. differentially expressed).

**Figure 4:**
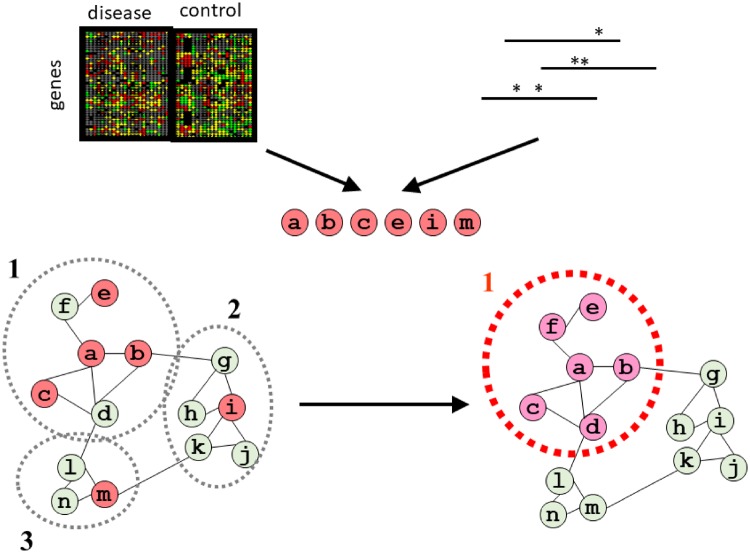
General strategy for discovering disease-related network modules. The starting point is an initial set of genes (*a, b, c, e, i* and *m*; red) related to the disease by multiple types of evidence: phenotypic evidence, such as differential expression (top left), or genotypic evidence, such as variants found in GWAS (top right). These genes are mapped to a biological network with three modules (1, 2 and 3; bottom left). A network propagation method detects module “1” as that related to the disease (i.e. enriched in the initial set of genes). Consequently, genes *f* and *d* are potentially linked to the disease, since they are involved in this module, while genes *i* and *m* could be discarded or ranked down in the prioritized list of variants.

The approaches for locating the topological module(s) associated with an initial set of genes are generally called “network diffusion” or “network propagation” [59, 60] (Fig. 4). These approaches locate network modules(s) “enriched” in the initial set of genes, generally by “propagating” some signal through the network edges from this initial set of nodes and recording the nodes at which the signal ends (Fig. 4). This can be done following different strategies. One possibility is to simulate “random walkers” that move in the network following the edges [61]. Algorithms developed to score the importance of web pages based on the network of hypertext links connecting them, such as Google’s PageRank, have been adapted to biological networks [62]. Different forms of graph kernels have also been used for this purpose [63], showing significant efficiency in measuring distances and functional relationships in genetic and protein networks [64, 65]. Physics-based approaches are also popular. These treat network connections (edges) as metal wires and simulate electric currents originated by applying voltage to sets of nodes, or diffuse heat starting from them. For example, HotNet [66] treats seed nodes as heat sources and network edges as “metal wires” able to transmit it. After simulating this heat diffusion for a while, the final set of “hot” nodes is reported as the resultant module. It is easy to see that heat would tend to confine to the topological module with most of initial nodes. Many of these approaches can be easily adapted to weighted and/or directed networks: e.g. by allowing a larger amount of signals to propagate through a “wider” edge, or by forcing random walker to move obeying the directions of edges. Similarly, they can be adjusted to work with multipartite and heterogeneous networks with different types of entities and relationships [49].

In some contexts, the modules detected from sets of mutated genes or those accumulating GWAS variants are called “genotypic modules,” as they come from genotypic evidences, whereas those detected from sets of genes with altered expression are termed as “phenotypic modules” [67]. If both sources of evidences are available for the same disease, they could map to different modules, one related to the genetic regulation of the processes/pathways associated to the disease, and another reflecting the altered pathways themselves. Under the assumption that both modules should be connected in some way, since genotypic alterations determine the observed phenotypic ones, some network propagation approaches use both genotypic and phenotypic sets of genes and “expand” them by including the genes required for connecting them [67].

## Software for locating disease-related modules

A variety of free software is available to interested users for performing “network propagation” with their datasets. These software are available in different models, from command-line tools to web interfaces, API services, etc. See Cowen *et al.* [60] for a detailed list of available programs.

Of special interest for nonexpert users is the possibility of using these approaches within Cytoscape (www.cytoscape.org), a widely used package for visualizing and manipulating biological networks [25]. This functionality is now integrated in the recent versions of Cytoscape (3.6 and above), and can be imported as plug-ins in previous versions [68]. This Cytoscape feature includes implementations of two of the network propagation approaches mentioned above: random walks and heat diffusion. To use this feature, the user must first select the initial set of nodes in Cytoscape, either in the network representation, via the table panel, using the “Select” tab/menu, or by any other selection mechanism. Then the user must go to the “Tools” menu and select “Diffuse.” After performing network propagation, two new attributes (data columns) are added to the nodes containing the scores produced by the two approaches. It is then possible to use other Cytoscape’s functionalities with these new attributes, e.g. selecting the nodes with the highest values (as potential detected modules) or coloring the nodes according to their scores. Additionally, a new “Results Panel” tab shows up with slider bars for automatically selecting top nodes according to both scores.

**Figure 5:**
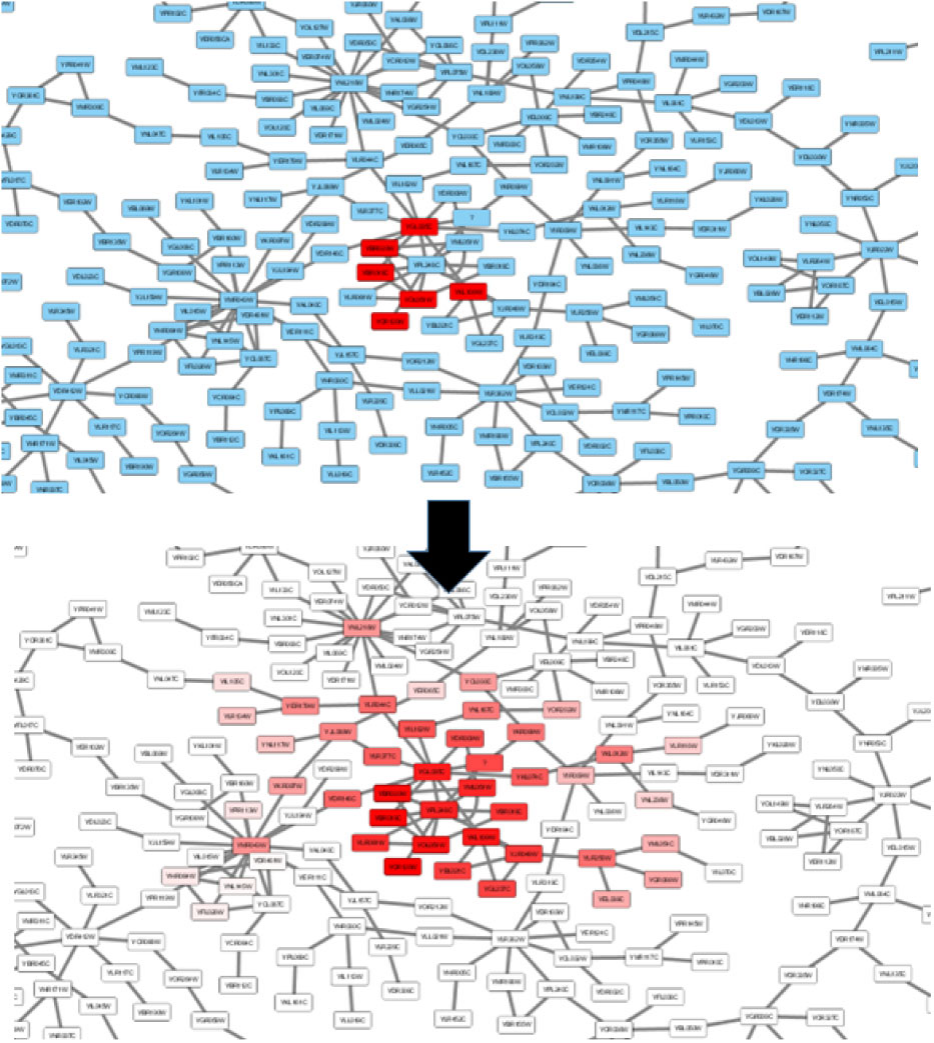
Example of network propagation using Cytoscape. Red nodes in the top panel are the initial set. Nodes in the bottom panel are colored according to their rank in the propagated signal (heat in this case). The network used is the “galFiltered” sample network that comes with Cytoscape’s distributions.

Figure 5 shows an example of network propagation performed with Cytoscape.

## Utility of disease-related modules

The overlap between topological modules, functional modules and disease-related modules, although not always perfect (Fig. 3), allows working under the scenario that “disease X is characterized by a malfunction/problem with biological pathway/function Y, associated with the network topological module Z.” This has multiple advantages with respect to using the initial sets of genes without considering their network context.

The detected module allows the identification of new genes potentially important/useful for the disease (e.g. “f” in Fig. 4, connecting “a” and “e”). These could be genes not originally identified due to experimental errors (“false negatives”) or simply genes not mutated or phenotypically altered but nevertheless important for understanding the disease and/or better markers or targets for treating it. For example, Ruffalo *et al*. [69] used network propagation to find genes previously known to be associated with cancer that nonetheless neither present mutations nor have altered expression.

On the other hand, this strategy allows us to discard genes (e.g. “i” in Fig. 4). This has implications for “variant prioritization” [70]: GWAS-like studies usually report many variants that have to be filtered/ranked. Network propagation approaches are routinely used for this [71]. Something similar happens with “copy number variation” (CNV) data associated with a disease: these genomic rearrangements involve large genomic regions, and not all the genes within these regions are necessarily causative. The pathological symptoms of CNVs vary depending on the genetic background, such as dosage-sensitive genes and recessive alleles located in the affected genomic region in each patient [72], making it challenging to discriminate between benign and pathogenic CNVs [73]. Within the many genes coded in a set of CNVs associated with a disease, those related to the disease are expected to cluster together in molecular networks, while the rest should be spread throughout the whole network. These “unrelated” genes could also be due to experimental errors (i.e. “false positives”).

Consequently, the final set of genes (refined by network criteria as explained above) could be a better “marker” for the diagnosis or prognosis of a disease. For example, it has been experimentally shown in some cases that the disease modules detected were better predictors of prognosis than any of the initial individual genes or combinations of them generated disregarding their network context [74, 75]. It was also shown that these network modules allow a better stratification of cancer patients into disease subtypes than the original set of genes [76].

Another benefit of inferring the network module(s) associated with a disease is to put that disease into a biological context: since topological modules are also functional modules, it is possible to associate a given disease with a functional pathway or a set of pathways (e.g. disease associated to biological process/pathway “1” in the example shown in Fig. 4) which could eventually provide additional information on pathology and even open new research avenues. This could be particularly important in the case of rare disorders.

Finally, putting a disease into a network context allows us to devise network-based strategies for treating it. For example, Lee *et al*. [77] detected a malfunctioning network module (signaling cascade in this case) associated with cancer and designed a rewiring strategy to generate a pathway with restored activity. In other cases, even if it was not possible to restore the pathway by rewiring, the authors managed to engineer a synthetic module to perform the function of a malfunctioning one [78].

As commented above, not only diseases are associated to network clusters but symptoms (patho-phenotypes/clinical signs) cluster in these networks as well [58]. Consequently, the same “network propagation” strategies can in principle be used to “redefine” a set of genes originally associated with a clinical sign using network information [79]. However, not all clinical signs cluster in compact modules. Several clinical and biological factors explain the variable performances of network-based prioritization approaches for clinical sign prediction [80]. These variable performances would unevenly impact the results of the approaches that perform network-based “gene prioritization” of genes found in a genomic study of undiagnosed patients based on their clinical signs [81–83].

## Other network-based approaches to human pathologies

Network-based approaches to study human pathologies are not restricted to the molecular networks described above (networks representing relationships between molecular entities). Many other disease-related phenomena have been modeled as networks and studied from that point of view. For example, networks representing drug-target and drug-drug relationships (e.g. based on chemical similarity, biological effect similarity, target(s) similarity, etc.), drug-disease associations, drug side effects, disease-disease associations or patient-patient relationships have been assembled and studied [84].

The “diseasome” is a network of diseases linked by common features [35]. Diseases can be linked if they share some characteristics, such as associated genes [47], microRNAs [85], functional linkages [86], protein localization [87], protein interactions [88], comorbidity patterns [89], signs and symptoms [90], or if their associated genes code for sequential reactions in metabolic pathways [91]. All these formalisms start from a bipartite graph representation (e.g. gene–disease associations), and derive a network projection of a single-nodetype (disease–disease associations). For that, two types of indices are used (i.e. to associate two diseases based on their own disease–gene associations): similarity indices and statistic-based indices (see [92]). These diseasome networks allow us to globally study relations between diseases from a global/systemic perspective. Some of these diseasomes are available online so that interested users can browse these disease connections (e.g. MalaCards [93], DiseaseConnect [94] and Orphan Disease Connections (ODCs) [95].

A tripartite network linking phenotypes with patients and genomic loci has been developed to identify novel genotype–phenotype relationships [96]. This network has been assembled from clinical data of thousands of patients with rare disorders collected by international consortia [97]. Mutations for many of these patients have been characterized genotypically and their pathological terms annotated using standard ontologies such as the Human Phenotype Ontology (HPO) [98].
